# About Bees

**Published:** 1882-11

**Authors:** 


### ABOUT BEES.

### HOW THEY LIGHT ON FLOWERS AND HOW THEY DO NOT FLY IN STRAIGHT LINES.

C. E. RIDDLER, of Canton, Mass., writes to the *Journal of Education* some highly interesting facts about the bee, obtained by long and patient personal observation. Following are extracts from his letter: "Bees, large and small, never alight on the top of the iris, but always in the same place, between one of the winged styles and one of the petals. I have watched hundreds of them. I have never seen an exception. Soon after alighting they force their bodies down as far as they possibly can into the very narrow space where the petal and style are very close together, breaking the pollen from the anthers above them, and in this place they extract the honey. I have seen them do this on the iris of the garden and on the *Iris Virginica* and the *Iris versicolor* of the meadows, and the process is the same, a bee alighting on one of the irids in my hand."

Mr. Riddler thinks that the office performed by the bee in making flowers productive is to brush the pollen from the anthers and convey it on its body directly into the ovary which it pierces. He thus upsets a well-known popular fancy:

"There is one popular delusion about bees, and that is that a 'bee-line' is a straight one. Let the following observation be considered: The hundreds of bees in the meadows, seeking honey of the iris, and going away laden with it, go in a series of delicately-curved lines, and not in straight ones. In the woods and fields their course is just the same. Sitting or reclining on a favorite knoll, where bees are continually flying about, I have seen them, day after day, at the rate of six or eight every minute, make a number of circles, large and small, around my head, and then fly off with great rapidity, not in straight lines for any considerable distance, and this, too, where there was plenty of room for them to fly a great distance in straight lines without any obstructions, but from side to side. in a kind of zigzag motion or curved lines.

"Again, passing along the street, there is a great buzzing in the trees, near by. It is a swarm of bees. They are directly overhead, and I stop and watch them; there's millions in it. They too fly a long distance within sight, not in straight lines, but in curves—majestic curves. Many of them fly swiftly from one side of the swarm to the other, but always in curves. Perhaps they are the

'mounted police,' and this may explain their curve-like motion, but it does not explain why the whole swarm moves thus.

"Once more: near the last day of the term a bee flies into the school-room. During its flight across the room, perhaps twenty-five or thirty feet, it changes its course four or five times, alights on a window-pane, makes several ineffectual attempts to climb its smooth surface, scents a rose on a neighboring desk, is on it in an instant (curves again), is unceremoniously thrust to the floor by the young lady owning the rose, and then in another instant makes a bee-line (that is such a line—curves here, too,—as a bee makes) for the open window, and is off.

"From these observations, a bee-line is not a mathematically straight one, any more than Court street, or some of the older streets of Boston, as they originally existed, were straight, any more than cow paths are straight, or the path of a squirrel climbing a tree to avoid the stone which the small boy is pretty apt to throw at him."

